# Shape‐Morphing in Oxide Ceramic Kirigami Nanomembranes

**DOI:** 10.1002/adma.202404825

**Published:** 2024-10-10

**Authors:** Minsoo Kim, Donghoon Kim, Mathieu Mirjolet, Nick A. Shepelin, Thomas Lippert, Hongsoo Choi, Josep Puigmartí‐Luis, Bradley J. Nelson, Xiang‐Zhong Chen, Salvador Pané

**Affiliations:** ^1^ Multi‐Scale Robotics Lab Institute of Robotics and Intelligent Systems ETH Zurich Tannenstrasse 3 Zurich 8092 Switzerland; ^2^ PSI Center for Neutron and Muon Sciences Paul Scherrer Institut Villigen 5232 Switzerland; ^3^ Department of Robotics & Mechatronics Engineering DGIST‐ETH Microrobotics Research Center Daegu Gyeong‐buk Institute of Science and Technology (DGIST) Daegu Republic of Korea; ^4^ Departament de Ciència de Materials i Química Física, Institut de Química Teòrica i Computacional Universitat de Barcelona Barcelona 08028 Spain; ^5^ Institució Catalana de Recerca i Estudis Avançats (ICREA) Pg. Lluís Companys 23 Barcelona 08010 Spain; ^6^ State Key Laboratory of Photovoltaic Science and Technology Shanghai Frontiers Science Research Base of Intelligent Optoelectronics and Perception, Institute of Optoelectronics, and International Institute of Intelligent Nanorobots and Nanosystems Fudan University Shanghai 200433 P. R. China; ^7^ Yiwu Research Institute of Fudan University Yiwu 322000 P. R. China

**Keywords:** ferroic nanocomposites, kirigami, microactuators, nanomembranes, stimulus responsive materials

## Abstract

Interfacial strain engineering in ferroic nanomembranes can broaden the scope of ferroic nanomembrane assembly as well as facilitate the engineering of multiferroic‐based devices with enhanced functionalities. Geometrical engineering in these material systems enables the realization of 3‐D architectures with unconventional physical properties. Here, 3‐D multiferroic architectures are introduced by incorporating barium titanate (BaTiO_3_, BTO) and cobalt ferrite (CoFe_2_O_4_, CFO) bilayer nanomembranes. Using photolithography and substrate etching techniques, complex 3‐D microarchitectures including helices, arcs, and kirigami‐inspired frames are developed. These 3‐D architectures exhibit remarkable mechanical deformation capabilities, which can be attributed to the superelastic behavior of the membranes and geometric configurations. It is also demonstrated that dynamic shape reconfiguration of these nanomembrane architectures under electron beam exposure showcases their potential as electrically actuated microgrippers and for other micromechanical applications. This research highlights the versatility and promise of multi‐dimensional ferroic nanomembrane architectures in the fields of micro actuation, soft robotics, and adaptive structures, paving the way for incorporating these architectures into stimulus‐responsive materials and devices.

## Graphical abstract

Multi‐dimensional multiferroic nanomembrane architectures with stretchability and shape reconfigurability are demonstrated. Complex 3‐D microarchitectures such as kirigami frames are fabricated using strain and geometrical engineering. Large mechanical deformation by stretching and dynamic shape‐morphing by electric stimuli are demonstrated. These findings open new avenues in the actuation of microscale 3‐D architectures, showcasing the potential of smart materials, micro robotics, and microelectromechanical systems.

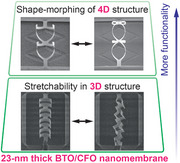

## 1 Introduction

The realization of 3‐D functional micro and nanostructures is essential for the advancement of miniaturized devices and systems with unique mechanical, magnetic, electronic, optical, acoustic, and thermal properties.^[^ ^1^, ^2^ ^]^ Strategically designed 3‐D structures can bring additional functionalities that do not exist in bulk materials and can significantly enhance their performance in a wide range of applications, including microelectromechanical system (MEMS) components,^[^ ^3^, ^4^ ^]^ electronics,^[^ ^5^, ^6^, ^7^ ^]^ biomedical devices,^[^ ^8^, ^9^, ^10^, ^11^, ^12^ ^]^ and energy storage solutions.^[^ ^13^, ^14^ ^]^ Several different techniques, such as 3‐D printing,^[^ ^15^, ^16^, ^17^, ^18^ ^]^ self‐assembly,^[^ ^19^ ^]^ template‐based deposition,^[^ ^9^, ^20^, ^21^, ^22^ ^]^ and material removal from bulk substrates^[^ ^23^, ^24^ ^]^ have been utilized to fabricate numerous 3‐D functional micro and nanostructures.^[^ ^25^, ^26^, ^27^ ^]^ Unfortunately, these techniques often fail to incorporate single crystalline or epitaxially grown high‐performance thin films primarily because of difficulties in materials synthesis.

Nanomembrane assembly offers key advantages in fabricating complex structures using high‐performance functional thin films (less than 100 nm thick).^[^ ^28^ ^]^ Physical vapor deposition or chemical vapor deposition followed by the subsequent etching of the sacrificial layer allows for the assembly of nanomembranes, accommodating a variety of functional materials such as semiconductors,^[^ ^29^, ^30^, ^31^ ^]^ graphene,^[^ ^32^, ^33^, ^34^ ^]^ metal dichalcogenides,^[^ ^35^, ^36^ ^]^ ferroic oxides,^[^ ^37^, ^38^, ^39^, ^40^ ^]^ gold,^[^ ^41^ ^]^ and hybrid composites.^[^ ^42^, ^43^ ^]^ Strain engineering in these nanomembranes can tune their intrinsic properties such as band structure, charge transport, electrical polarization, and magnetism,^[^ ^44^, ^45^, ^46^, ^47^, ^48^ ^]^ and also engineer their complex 3‐D nano architectures,^[^ ^49^, ^50^, ^51^ ^]^ providing extraordinary device configurations and properties.^[^ ^52^ ^]^ Furthermore, kirigami patterns, inspired by the Japanese art of paper cutting offer an even broader spectrum of design possibilities due to the paper‐like mechanical behavior of these nanomembranes. Recent innovations, such as graphene kirigami and origami,^[^ ^53^, ^54^ ^]^ as well as the utilization of gold nanosheets in kirigami designs for adjusting optical chirality,^[^ ^55^ ^]^ underscore the emerging potential of material structure engineering to unlock unconventional properties.

**Figure 1:**
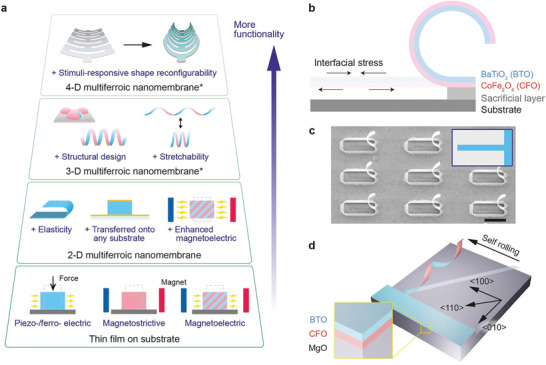
a) Schematic diagram emphasizing how multi‐dimensional multiferroic nanomembrane architectures enable the integration of advanced functionalities by exploiting mechanical, electrical, and magnetic properties. 4‐D nanomembranes refer to 3‐D nanomembranes that can change their shape in response to external stimuli. ^*^This research focuses on the development of nanomembrane architectures in 3‐D and 4‐D. b) Illustration of the mechanism for creating nanomembrane 3‐D architectures using bilayer nanocomposites of barium titanate (BaTiO_3_, BTO) and cobalt ferrite (CoFe_2_O_4_, CFO). The interfacial stress induced by the lattice mismatch causes the BTO/CFO nanomembrane to roll upon itself when detached from the substrate. c) Scanning Electron Microscope (SEM) image showcasing a self‐rolled BTO/CFO 3‐D arc architecture originating from a stripe pattern. (Scale bar: 10 µm). Inset: illustration depicting the top view of a BTO/CFO stripe prior to surface detachment. d) Formation mechanism behind helical structures from a diagonal stripe pattern of the BTO/CFO nanomembrane driven by anisotropy in Young's modulus.

Ferroic or multiferroic nanomembranes have been shown to exhibit exceptional mechanical properties such as stretchability, superelasticity,^[^ ^56^ ^]^ and shape‐memory behaviors,^[^ ^38^ ^]^ while maintaining or exceeding their bulk properties such as ferro‐/piezo‐ electric, ferromagnetic, and magnetoelectric properties (**Figure** **1**a).^[^ ^39^, ^57^, ^58^, ^59^, ^60^ ^]^ Single crystalline and epitaxial ferroic nanomembranes can be assembled by detaching films from the substrate where the film is grown. These nanomembranes can then be transferred onto various substrates, such as Si, using van der Waals interactions, facilitating the integration of ferroic oxides into semiconductor‐based device configurations. Strain engineering in ferroic nanomembranes has been mainly focused on substrate transfer^[^ ^61^ ^]^ or stretching via flexible substrates^[^ ^39^, ^46^, ^60^ ^]^ rather than geometric engineering. In this regard, the inclusion of interfacial residual stress in the ferroic nanomembranes via heteroepitaxial layering broadens the scope of ferroic nanomembrane assembly and enables the creation of complex 3‐D structures,^[^ ^62^ ^]^ offering ample opportunities to explore enhanced physical properties.^[^ ^38^, ^39^, ^63^ ^]^

Here, we develop multi‐dimensional complex multiferroic structures by capitalizing on interfacial strain engineering and advanced lithography techniques in order to introduce a broader range of functionalities (3‐D and 4‐D nanomembranes, shown in Figure 1a), including exceptional mechanical stretchability and shape reconfigurability. Using the interfacial stress between lattice mismatched layers, we design various self‐rolled up 3‐D architectures including arcs, helices, diamond‐kirigami, and ribbon‐kirigami frames. Under in situ nanomechanical tensile testing, these structures are able to undergo large deformations and recovery. Furthermore, we demonstrate the bending actuation and shape‐morphing of diamond and ribbon‐kirigami frames under electron beam irradiation, providing stimulus‐responsive shape reconfiguration possibilities (4‐D printing). Our research highlights the versatile potential of these innovative 3‐D ferroic nanomembrane architectures in stimulus‐responsive micro actuation and micro robotic applications.

## 2 Results

### 2.1 Formation of 3‐D Architecture

**Figure 2:**
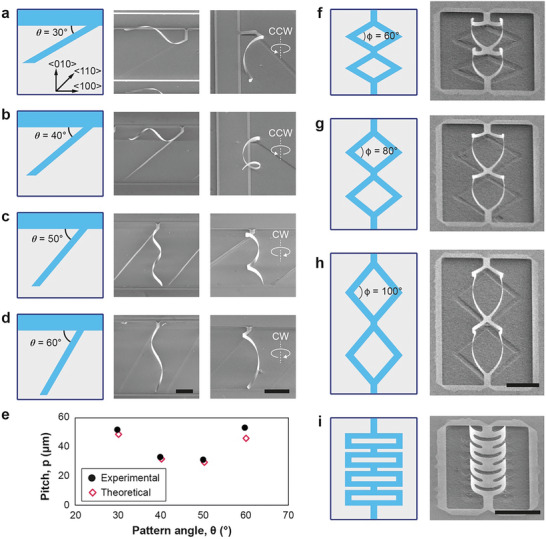
Formation of various 3‐D architectures depending on the pattern designs. a–d) Helices with pattern angles of 30°, 40°, 50°, and 60° for each design, respectively. Each image set includes a pattern design along with SEM images, featuring both a top view and a 45° tilted view. e) Change in helical pitch length as a function of pattern angles (θ). f–h) Diamond‐kirigami 3‐D frames with pattern angles (ϕ) of 60°, 80°, and 100° for each design, respectively. Each image set includes a pattern design and a SEM image (45° tilted view). i) A ribbon‐kirigami 3‐D frame. The image set includes a pattern design and a SEM image (45° tilted view). The crystalline axis described in the pattern design of (a) also corresponds to the pattern designs of (b–d) and (f–i). Scale bars indicate 10 µm.

Figure 1b demonstrates the interfacial strain‐engineered ferroic nanomembrane assembly process utilizing epitaxial BaTiO_3_ (BTO, 8 nm)/CoFe_2_O_4_ (CFO, 15 nm) bilayers grown on a MgO (001) substrate (Figure S1, Supporting Information), which shows clear magnetoelectric behavior (Figure S2, Supporting Information). The lattice mismatch between BTO and CFO, which corresponds to ≈5%, leads to interfacial stress that causes the bilayer to roll upon release from the substrate eventhough the BTO layer is partially relaxed. High‐resolution scanning transmission electron microscopy (HR‐STEM) images of the as‐deposited BTO/CFO layer further show that the misfit strain causes the BTO layer to be stretched near the interface and relax along its thickness (Figures S3 and S4, Supporting Information). Using photolithography, and dry and wet etching (detailed process flow in Figure S5, Supporting Information), we fabricated various BTO/CFO nanomembrane 3‐D architectures. Stripe patterns aligned parallel to the [100] crystalline axis give rise to arc‐shaped rolls (Figure 1c). Note that bilayers that are not epitaxially grown or that are grown fully relaxed were unable to form freestanding nor self‐rolled structures due to the lack of driving force for the initial shape deformation, i.e., interfacial stress (Figure S6, Supporting Information). Additionally, diagonal stripes with certain angles to the [100] axis form helical shapes (Figure 1d),^[^ ^38^ ^]^ influenced by both the anisotropic Young's modulus of the BTO/CFO nanomembrane (higher elasticities in [100]/[010] directions than [110] direction)^[^ ^64^ ^]^ and preferential etching direction of the MgO substrate (along [100]/[010] direction).^[^ ^31^ ^]^ The preferential rolling direction toward the [100] or [010] direction provides control over the chirality and the pitch length of the helices. Diagonal patterns with angles (θ) of 30° and 40° from the [100] axis yield counter‐clockwise (CCW) chirality (**Figure** **2**a,b), and angles of 50° and 60° produce clockwise (CW) chirality as the diagonal stripes roll toward the [010] axis (Figure 2c,d). The helical formation also aligns with its finite element method simulation result (Figure S7, Supporting Information). The observed opposite chirality in the helices, with pitch length varying from 31 to 53 µm, demonstrates the symmetry of the rolling relative to the pattern angle of 45° ([110] direction), as shown in Figure 2e. The pitch length is theoretically determined using Equation (1),

(1) p=2πRtanθ

where R represents the radius of the helix (measured from Scanning Electron Microscope (SEM) images, Figure S8, Supporting Information) and θ is the pattern angle. With a 45° pattern angle, helical structures showed random chirality due to a reduced etching rate of the MgO substrate along the [110] direction (Figure S9, Supporting Information).

Geometric engineering in BTO/CFO nanomembranes further enables the configuration of Kirigami‐inspired designs. This offers enhanced mechanical stress and strain durability, which are not achievable in the nanomembrane itself.^[^ ^2^, ^53^, ^65^, ^66^ ^]^ We introduced four different kirigami patterns, including diamond‐kirigami frames with diamond pattern angles (ϕ) of 60°, 80°, and 100° (Figure 2f–h, Figure S10, Supporting Information), and a ribbon‐kirigami frame (Figure 2i). These kirigami 3‐D frames also align with the results of their finite element method simulations (Figures S11 and S12, Supporting Information). In contrast to helices, these designs roll up without forming any spirals or twists, as their symmetric nature results in zero net torque. The out‐of‐plane deformations caused by rolling up are evaluated by bending angles, under the assumption that the rolled‐up structures form circular cross‐sections (Figure S13, Supporting Information). The bending angles for self‐rolled diamond‐frame structures with diamond pattern angles of 60°, 80°, and 100° are estimated to be 259 ± 7°, 248 ± 5°, and 255 ± 2°, respectively. These values correspond to the radius of curvature of 4.0 ± 0.1, 3.9 ± 0.1, and 4.1 ± 0.1 µm, respectively. The ribbon‐kirigami design exhibits a bending angle of 138 ± 3° and a radius of curvature of 4.2 ± 0.1 µm.

### 2.2 Stretchability of Kirigami Frames

**Figure 3:**
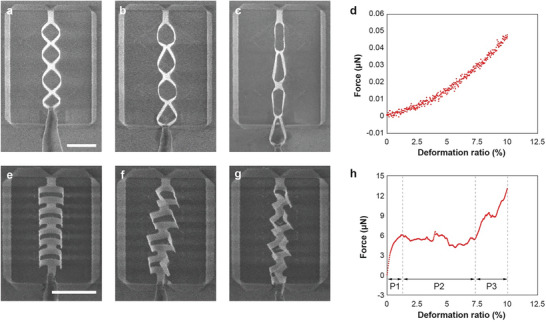
Demonstration of large tensile deformation ratios in a–d) an 80° diamond‐kirigami frame and e–h) a ribbon‐kirigami frame. SEM images of the diamond‐kirigami frame show (a) the initial state, (b) stretching to 10% of sample length (4 µm of displacement), and (c) stretching to 34% of sample length (13 µm of displacement, just before fracture). (d) Force‐displacement curve of the diamond‐kirigami frame during the deformation from (a) 0 µm to (b) 4 µm. SEM images of the ribbon‐kirigami frame display (e) the initial state, (f) stretching to 10% of the sample length (2 µm of displacement), and (g) stretching to 20% of the sample length (4 µm of displacement, just before fracture). (h) Force‐displacement curve of the ribbon‐kirigami frame under the deformation from (e) 0 µm to (f) 2 µm (P1: pre‐deformation, P2: plateau, P3: divergent regions). Scale bars indicate 10 µm.

**Figure** **3** illustrates the large stretchability of kirigami frames through in situ nanomechanical tensile testing. Upon in‐plane extension, both in‐plane and out‐of‐plane deformations were observed across all kirigami frames, leading to significant deformations. The 80° diamond‐kirigami frame exhibited ≈34% relative displacement prior to fracture (Figure 3a–c), while the ribbon‐kirigami frame withstood ≈20% relative displacement (Figure 3e–g). The force‐deformation curves for diamond‐kirigami (Figure 3d) and ribbon‐kirigami frames (Figure 3h; Video S1, Supporting Information) exhibit distinct tensile behaviors. Compared to the helices,^[^ ^38^ ^]^ diamond‐kirigami frames were stretched easily even with a small force applied (tens of nN, Figure 3d). In contrast, the ribbon‐kirigami structure endured large force variation during the deformation (two orders of magnitude higher than the diamond‐kirigami structure, Figure 3h). Furthermore, the force‐displacement curve for the ribbon‐kirigami structure exhibits three distinct stages, namely, the pre‐deformation (P1), the plateau (P2), and the divergent regions (P3). For displacements below 1%, linear deformation is observed, indicating that the kirigami frame deforms without any buckling and the mechanical characteristics are governed by Young's modulus. Upon stretching, the plateau region develops, corresponding to the buckling and out‐of‐plane motion of the structure.^[^ ^67^ ^]^ Finally, when the deformation ratio exceeds 7.5%, the buckled structure hardens, and out‐of‐plane deformation no longer occurs, resulting in divergent force‐displacement behavior. This behavior is consistently observed in repeated experiments (Figure S14, Supporting Information). The difference in the mechanical response in diamond‐ and ribbon‐kirigami frames could be attributed to the presence of the buckling.^[^ ^67^, ^68^ ^]^ Unlike the ribbon‐kirigami, diamond‐kirigami frames exhibit continuous motion without any buckling under tensile loads (Video S2, Supporting Information).

### 2.3 Electric Field‐Responsive Shape‐Morphing of Kirigami Frames

**Figure 4:**
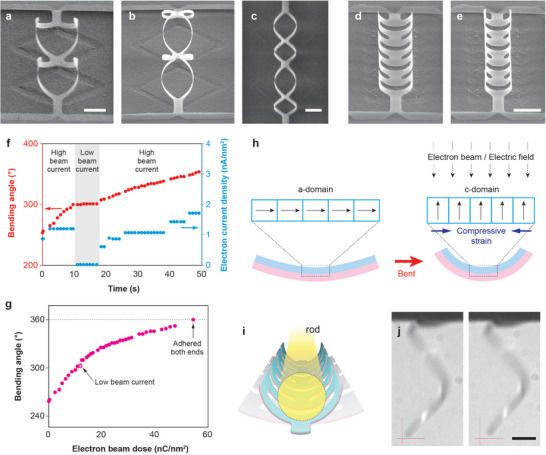
Electrically stimulated shape‐morphing of BTO/CFO nanomembrane 3‐D architectures. a‐b) Shape‐morphing of an 80° diamond‐kirigami frame (45° titled view). c) Both ends of the diamond‐kirigami frame adhere to each other (top view). d‐e) Shape‐morphing of a ribbon‐kirigami 3‐D structure (45° titled view). f) Change in bending angle over time is primarily influenced by electron beam current density. g) Bending angle variation as a function of electron beam dose, with hollow data points indicating low beam current (referenced in f). h) Schematic diagram illustrating the shape‐morphing mechanism. i) Illustration showing that shape‐morphing behaviors can serve as actuation mechanisms for microgrippers. j) Shape‐morphing of a helix induced by the application of an electric field (+ 30 V at the tip). The red cross serves as a reference for shape change. Scale bars indicate 5 µm.

These 3‐D architectures of BTO/CFO nanomembranes undergo stimulus‐responsive shape reconfiguration. When exposed to an electron beam, BTO/CFO kirigami frames change shape (**Figure** **4**a–e) by altering the roll‐up bending angle (Figure S13, Supporting Information). Partial electron beam exposure resulted in shape reconfiguration only in the exposed region (Figure S15; Video S3, Supporting Information), indicating that electron beam irradiation is the main cause of shape reconfiguration. The bending angle in Kirigami frames was significantly influenced by the beam current and exposure time (Figure 4f). High electron beam current density consistently increased the bending angle, whereas low beam current resulted in minimal changes. Upon deactivating (switching off) the electron beam, the structure gradually returned to its original equilibrium state (Figure S16, Supporting Information). Overall, the bending angle asymptotically increases with the electron beam dose (Figure 4g). In BTO/CFO nanomembranes, electron beam exposure shifts the stress‐strain balance state, possibly due to i) ferroelectric domain rotation (Figure 4h),^[^ ^38^, ^69^, ^70^, ^71^, ^72^ ^]^ ii) heating,^[^ ^73^, ^74^ ^]^ or iii) Coulomb interaction within the film.^[^ ^75^ ^]^ The most probable origin of stress‐strain balance shifting among these is ferroelectric domain rotation, supported by estimating the radius of curvature. The change in the radius of curvature in a bilayer is defined using Equation (2),

(2) $$\frac{1}{R}-\;\frac{1}{R_{0}}=\frac{3}{2m}\;\frac{\varepsilon }{h}$$

where *R*, *R*_0_, ε, and *h* are the final radius of curvature, the initial radius of curvature, the misfit strain, and the total thickness, respectively. $m=1+{\left(E_{1}h_{1}^{2}-E_{2}h_{2}^{2}\right)}^{2}/\left(4E_{1}h_{1}E_{2}h_{2}h^{2}\right)$ is a dimensionless coefficient, where *E*_1_ and *E*_2_ are the Young's modulus of each layer and *h*_1_ and *h*_2_ are the thickness of each layer.^[^ ^76^ ^]^ As misfit strain from the mismatch lattice parameter (*a*) between BTO/CFO layers can be relaxed, misfit strain can be defined through Equation (3) by introducing loss factor (ζ).^[^ ^77^ ^]^

(3) $$\varepsilon_{domain}=\zeta \;a$$

The change in the mismatch parameter (i.e., ≈1% by domain rotation a‐ to c‐domain) results in a change in misfit strain of ≈0.1%, described in Equation (4). It is assumed that the loss factor is not changed.

(4) $$\Delta \;\varepsilon_{domain}=\zeta \;\Delta a$$

As a result, the theoretical radius of curvature estimated by Equation (2) is similar to the experimentally measured radius of curvature after the bending. On the other hand, the temperature increase caused by the electron beam conditions used in this experiment is negligible (details in Supporting Information) and cannot achieve the same level of bending through thermal expansion.^[^ ^78^, ^79^ ^]^

**Figure 5:**
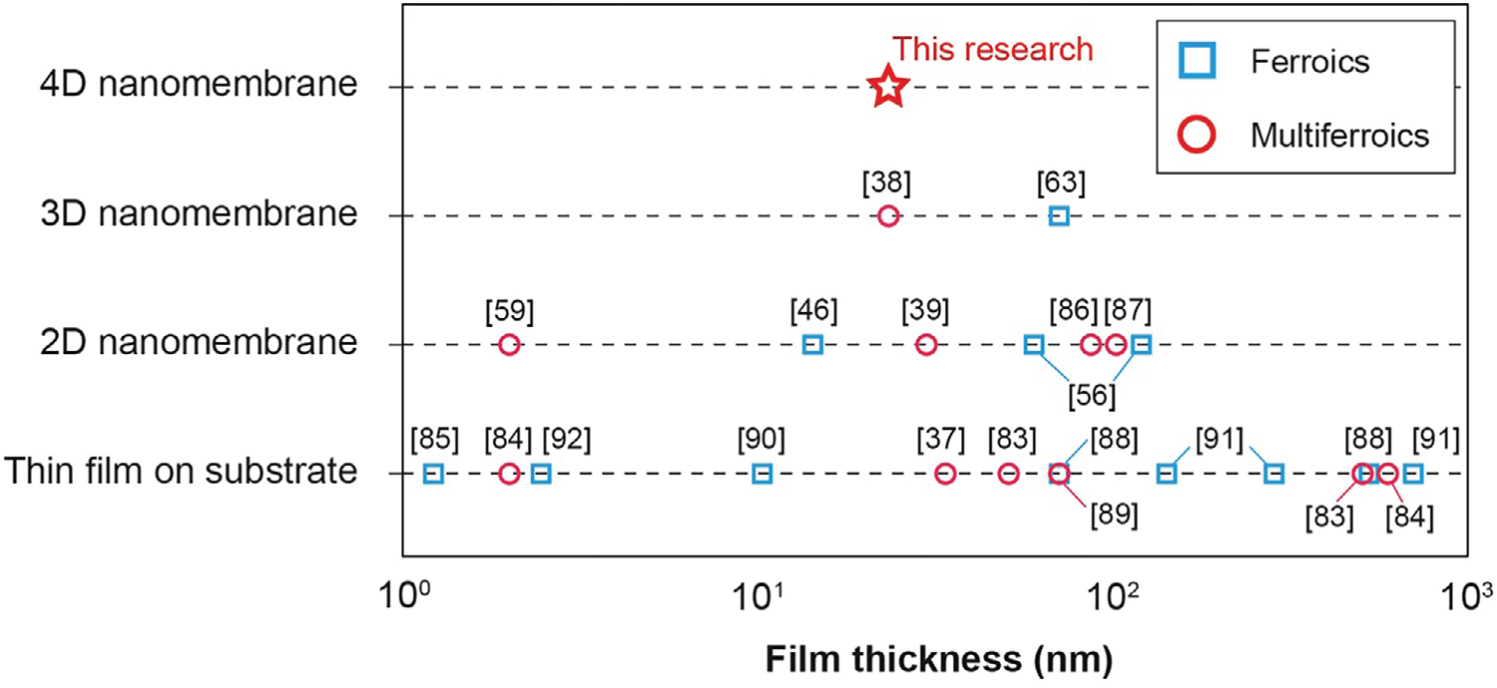
Comparison of ferroic and multiferroic nanomembrane multi‐dimensional architectures.^[^ ^37^, ^38^, ^39^, ^46^, ^56^, ^59^, ^63^, ^83^, ^84^, ^85^, ^86^, ^87^, ^88^, ^89^, ^90^, ^91^, ^92^ ^]^

As the bending angle approaches 360°, the edges of the kirigami frames adhere to each other abruptly, most probably from van der Waals or Coulomb interaction (Figure 4c; Video S4, Supporting Information).^[^ ^75^ ^]^ These grasping‐like bending motions could be suitable for electrically actuated microgrippers (Figure 4i). The shape reconfigurability of BTO/CFO nanomembranes was also tested by applying direct electric fields outside the SEM. We attached the BTO/CFO helix onto a tungsten wire, where the electric field can be applied to the tungsten tip (experimental setup in Figure S17, Supporting Information). With the application of a low voltage (+ 30 V), the movement of the helix was observed (Figure 4j; Video S5, Supporting Information). When we put the tungsten tip above the kirigami frames and applied the low voltage, we also observed shape morphing in the kirigami frames. (Figure S18, Supporting Information). Our observation showcases the opportunities for realizing 4‐D nanomembrane architectures, inspired by 4‐D printing technologies where the materials are initially fabricated in 3‐D, but can reconfigure their shapes in response to external stimuli such as temperature, chemical reactions, electric fields, or magnetic fields (**Figure** **5**).^[^ ^80^, ^81^, ^82^ ^]^

## 3 Conclusion

In this study, we developed BTO/CFO nanomembrane 3‐D architectures by leveraging the unique superelasticity and strain‐coupled ferroelectric domain rotation in multiferroic BTO/CFO heterostructures. Capitalizing on photolithography and substrate etching techniques, we created complex 3‐D ferroic nanomembrane architectures, including helix, diamond‐kirigami, and ribbon‐kirigami designs, which demonstrate remarkable mechanical deformation capabilities (i.e., stretchability). This feature underscores the mechanical robustness and flexibility of our designs, highlighting their potential in applications that require extensive deformation, such as electronic skins. Furthermore, the application of electric fields and electron beam exposure has yielded promising results in dynamic shape reconfiguration and actuation capabilities. Specifically, the bending behaviors observed in the kirigami structures under electron beam stimulation provide an opportunity for the development of electrically actuated microgrippers and other micromechanical devices. These findings highlight the potential of BTO/CFO kirigami frames to revolutionize the field of stimulus‐responsive materials and 4‐D architectures, where materials exhibit time‐dependent shape changes in response to external stimuli. Our research sets the stage for future breakthroughs in smart materials and devices, creating opportunities for applications in micro actuation, soft robotics, and adaptive structures. Exploring diverse material combinations within the 3‐D architecture of nanomembrane assembly can further augment functionalities (e.g., magnetoelectric effect), extending the range of applications well beyond MEMS devices to encompass the medical, photonics, and energy storage domains.

## 4 Experimental Section

### Fabrication of BTO/CFO Nanomembranes

Epitaxial BTO/CFO (001) bilayers were deposited on the MgO (001) substrates using pulsed laser deposition (PLD). A 15 nm‐thick CFO layer was deposited at 550 °C, 10 mTorr oxygen partial pressure, and laser parameters of 5 Hz, 1.8 J cm^−2^. Subsequently, an 8 nm‐thick BTO layer was deposited at 750 °C, 200 mTorr oxygen partial pressure with 4 Hz, 1.2 J cm^−2^ laser.^[^ ^38^ ^]^ Subsequently, the films were patterned to form microstructured designs using photolithography and Ar‐ion etching. After the chemical etching of the MgO substrate with the saturated sodium bicarbonate solution, the sample was dried using the critical point dryer.

### In Situ Nanoscale Mechanical Tensile Test

Tensile tests were performed under a scanning electron microscope (SEM, Nova NanoSEM 450, FEI Company) equipped with a nanomechanical testing system (FT‐NMT03, Femtotools AG) at room temperature. BTO/CFO nanomembrane kirigami frames were affixed to the force sensor probe tip with an SEM‐compatible glue (SEMGLU, Kleindiek Nanotechnik GmbH). The force was measured at a 100 Hz sampling rate under a tensile loading‐unloading rate of 1 µm s^−1^ using a force sensor (model FT‐S200). This sensor is equipped with a tungsten probe tip, featuring a radius of less than 0.1 µm, a force range limit of ± 200 µN, and a resolution of 0.5 nN.

### Electrical Stimulus Responsive Shape‐Morphing

The BTO/CFO nanomembrane 3‐D architectures were stimulated using an electron beam (acceleration voltage: 3 kV) in a SEM at room temperature. The beam dose was adjusted by changing the magnification to either enlarge or reduce the field of view. The beam dose was calculated based on the beam current and the area of the field of view. To demonstrate the strain relaxation and shape recovery of the deformed structures, the electron beam irradiation was halted for more than 10 min. Furthermore, shape‐morphing was achieved through electrical stimulation at low voltage, performed in a probe station setup equipped with a microwire (Goodfellow). This microwire, made of 99.95% tungsten and with a diameter of 15 µm, was connected to a power supply, and 30 V was applied intermittently by toggling the power on and off.

## Conflict of Interest

The authors declare no conflict of interest.

## Supporting information



Supporting Information

Supplemental Video 1

Supplemental Video 2

Supplemental Video 3

Supplemental Video 4

Supplemental Video 5

## Data Availability

The data that support the findings of this study are available from the corresponding author upon reasonable request.
