# Supplementary material for: Shape‐Morphing in Oxide Ceramic Kirigami Nanomembranes
Source: Adv Mater. 2024 Oct 10;36(47):2404825. doi: 10.1002/adma.202404825 (PMC11586819; doi:10.1002/adma.202404825)
Supplement: Supplementary file 1 — Supporting Information [file ADMA-36-2404825-s001.docx]

Supporting Information

Shape-Morphing in Oxide Ceramic Kirigami Nanomembranes

Minsoo Kim*, Donghoon Kim, Mathieu Mirjolet, Nick A. Shepelin, Thomas Lippert, Hongsoo Choi, Josep Puigmartí-Luis, Bradley J. Nelson, Xiang-Zhong Chen*, Salvador Pané*

**List of supporting videos**

**Supporting video S1.** Tensile testing of the ribbon-kirigami frame.

**Supporting video S2.** Stretching the diamond-kirigami frame (10× play speed).

**Supporting video S3.** Partial deformation of the diamond-kirigami frame in SEM (4× play speed).

**Supporting video S4.** Full shape-morphing of the diamond-kirigami frame in SEM (5× play speed).

**Supporting video S5.** Shape-morphing of the helix by the application of electric fields.

**Estimation of temperature change by electron beam heating**

The maximum temperature ($\theta_{max}$) due to electron beam irradiation is estimated by:

$$\theta_{max}=\frac{{V_{a}}/\kappa}{\pi^{1/2}}\frac{I_{p}}{d}$$

where $V_{a}$, $I_{p}$, $\kappa$, and $d$ are the acceleration voltage (3 kV), the beam current (<30 pA), the thermal conductivity of the material (4 Wm^-1^K^-1^), and the beam diameter (>20 nm), respectively. The maximum estimated value is around 1°C. However, a temperature of over 100°C is required if the different thermal expansion of layers is major origin of the bending.

**Finite element modeling of 3-D architecture formation**

A FEM software (COMSOL Multiphysics® 6.2) estimated the 3-D architecture formation of helical structures, diamond-kirigami 3-D frames, and a ribbon-kirigami 3-D frame. The material properties used in the simulation are described in Table S1. Thermal expansion was used to simulate the lattice parameter mismatch. We assigned different thermal coefficients according to the axis: ~0.4% along the [100] axis and ~0.1% along the [010] and [001] axes to reflect the preferable self-scrolling direction. The thermal strain was set to 0.6% for helices and 0.4% for kirigami structures. The boundary conditions were assigned to one end as a fixed condition. Sweep functions were used for precise estimation of large deformations. Figure S7, S11, and S12 shows simulation results corresponding to the experimental results in Figure 2.

**Table S1.** Material properties used in FEM simulation.

| Material | Property | Value |
| --- | --- | --- |
| Barium titanate | Density | 6020 kg m^-3^ |
|  | Young’s modulus | 177 GPa |
|  | Poisson’s ratio | 0.22 |
| Cobalt ferrite | Density | 5250 kg m^-3^ |
|  | Young’s modulus | 181 GPa |
|  | Poisson’s ratio | 0.25 |

**
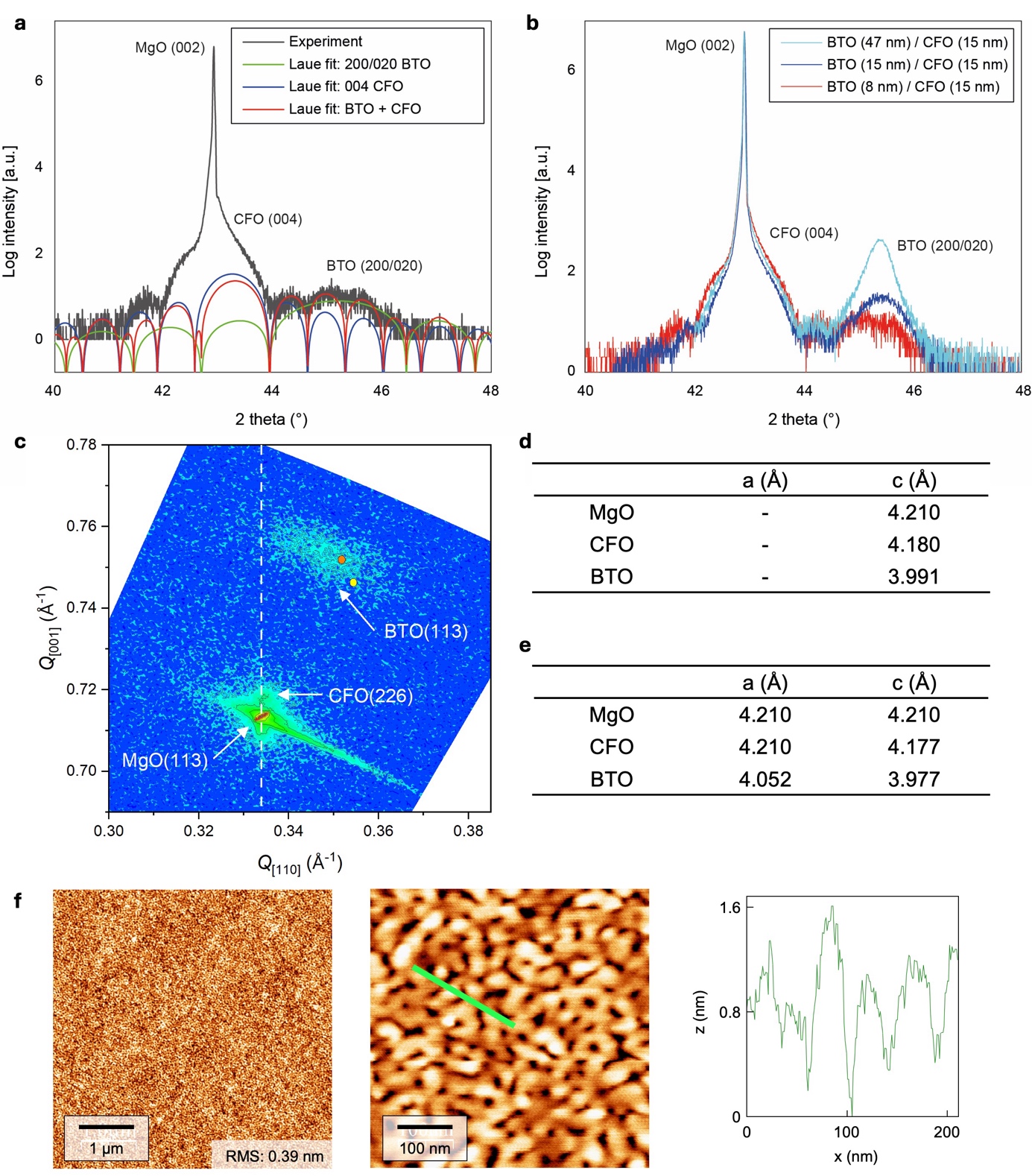
**

**Figure S1.** Epitaxial growth of BTO/CFO bilayer thin films. (a) Theta-2theta scan of the BTO (8 nm)/CFO (15 nm) epitaxial thin film grown on MgO (001) substrate. (b) Theta-2theta scans of different thickness of BTO layers. (c) Reciprocal space mapping of BTO (8 nm)/CFO (15 nm) bilayers. The orange and yellow dots indicate the reflections for the unstrained BTO crystal, corresponding to the a-domain (with lattice parameters a = 4.03 Å, c = 3.99 Å) and the c-domain (with lattice parameters a = 3.99 Å, c = 4.03 Å), respectively. The extracted cell parameters from (d) theta-2theta scan and (e) RSM. (f) Atomic force microscopy (AFM) images reveal that the BTO (8 nm)/CFO (15 nm) bilayer surface exhibits low root-mean-square (RMS) roughness. The surface profile, obtained from the highlighted line, indicates a low peak-to-valley distance.


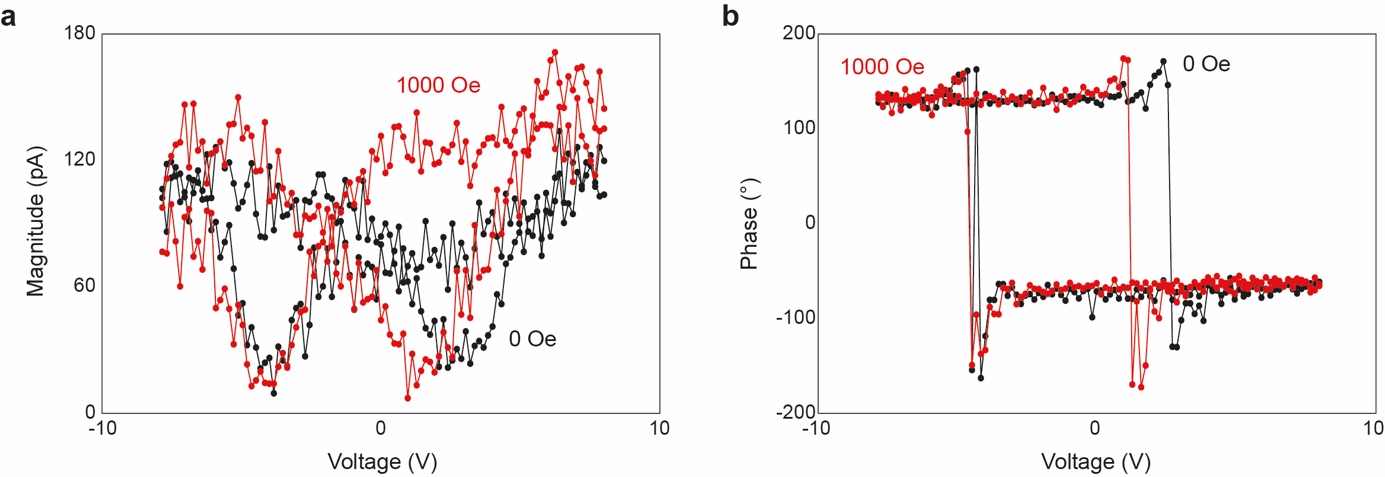


**Figure S2.** Magnetoelectric effect of BTO/CFO bilayer. Piezoresponse force microscopy loops. (a) Magnitude. (b) Phase.

*
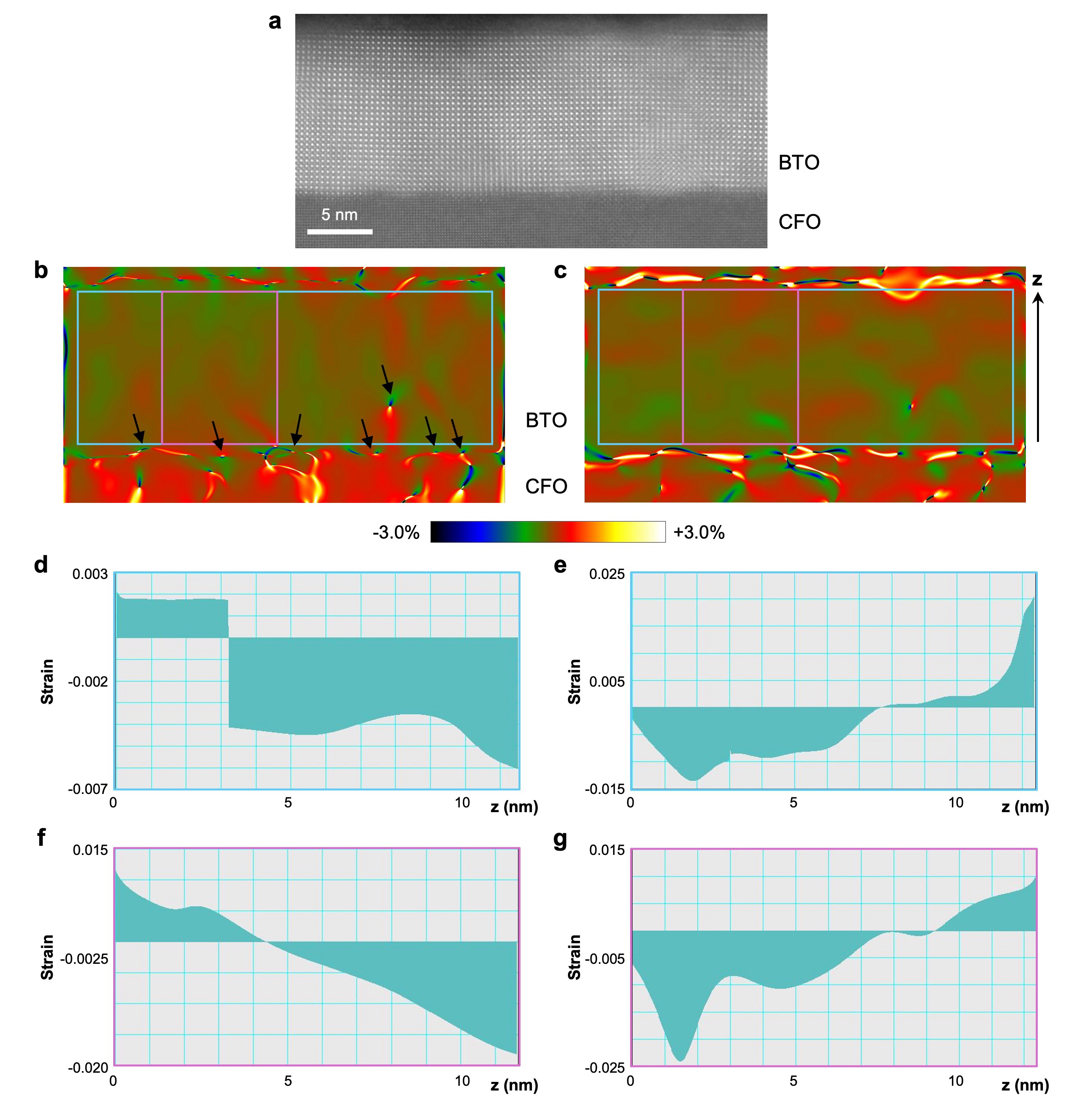
*

**Figure S3.** (a) High-angle annular dark-field scanning transmission electron microscopy (HAADF-STEM) image of BTO/CFO bilayer thin film on MgO substrate and corresponding geometric phase analysis of (b) in-plane strain and (c) out-of-plain strain in BTO layer. Average strain profiles of indicated areas are extracted for (d) large blue rectangular and (f) small purple rectangular areas of the in-plane strain, and (e) large blue rectangular and (g) small purple rectangular areas of the out-of-plane strain.


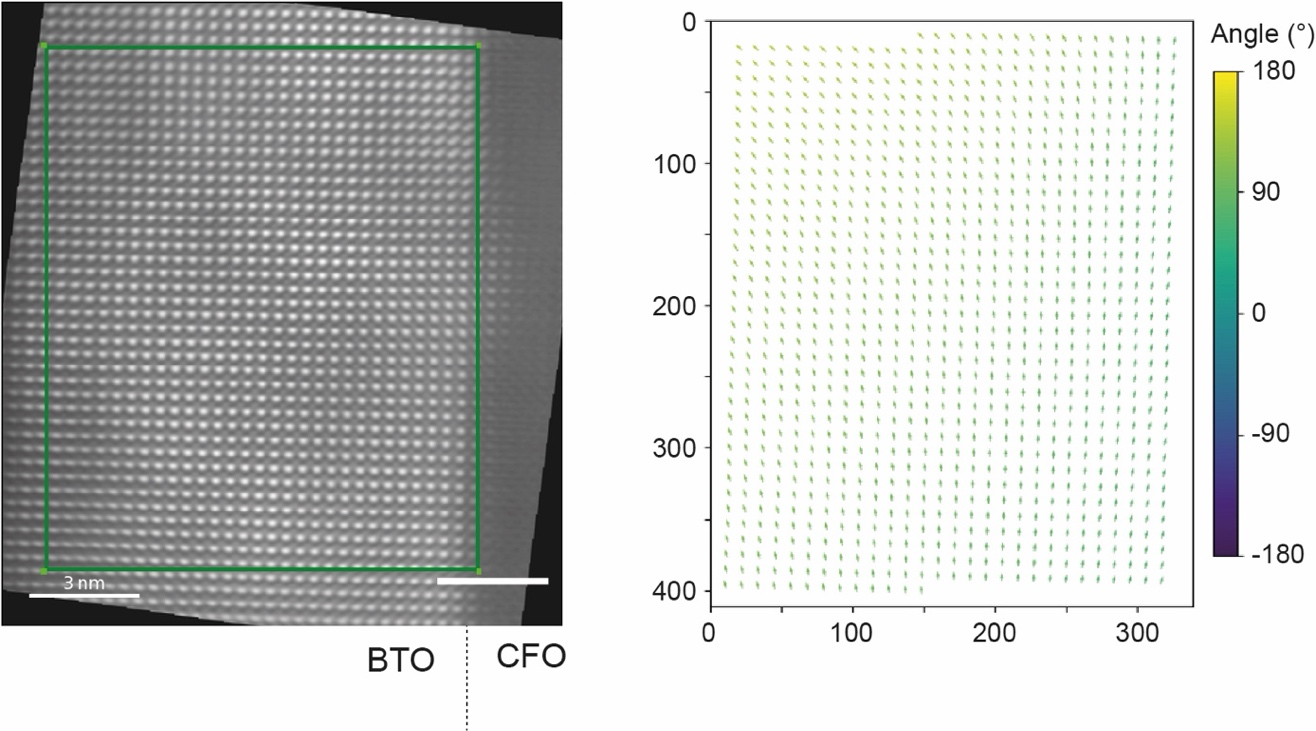


**Figure S4.** (Left) HR-STEM image and (right) the corresponding Ti-ion displacement analysis. The BTO layer exhibits in-plane domains near the interfaces (Scale bar: 3 nm).


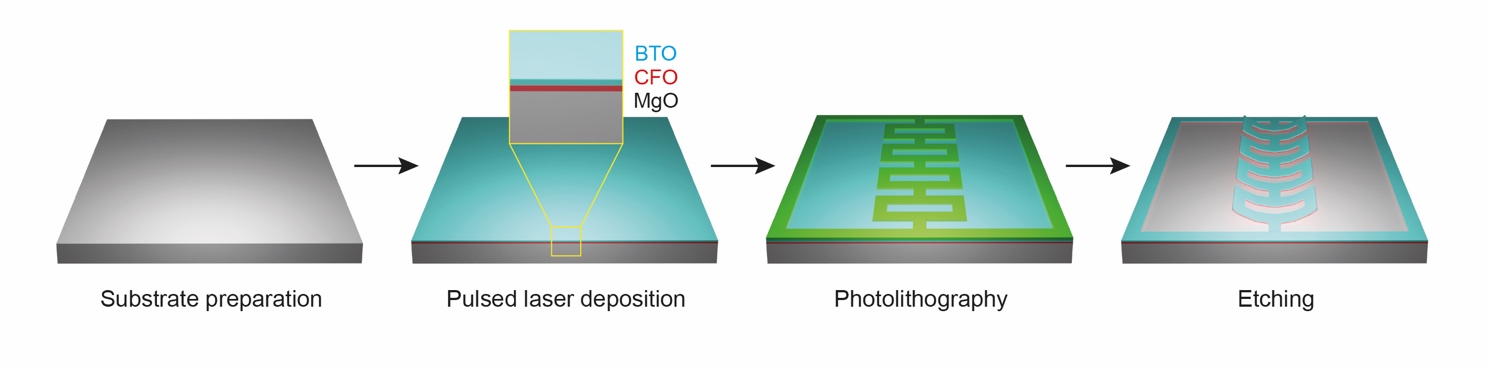


**Figure S5.** Fabrication process of 3-D BTO/CFO nanomembrane architectures.


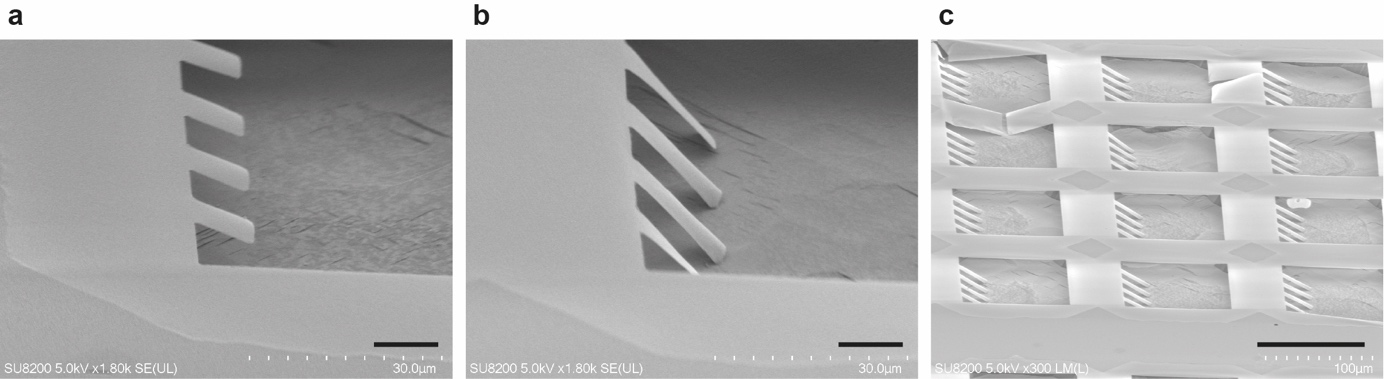


**Figure S6.** SEM images showing that bilayers with fully relaxed BTO layer failed to form self-rolled arc-shaped structures. Scale bars in (a) and (b) indicate 10 µm, and in (c) indicate 100 µm.


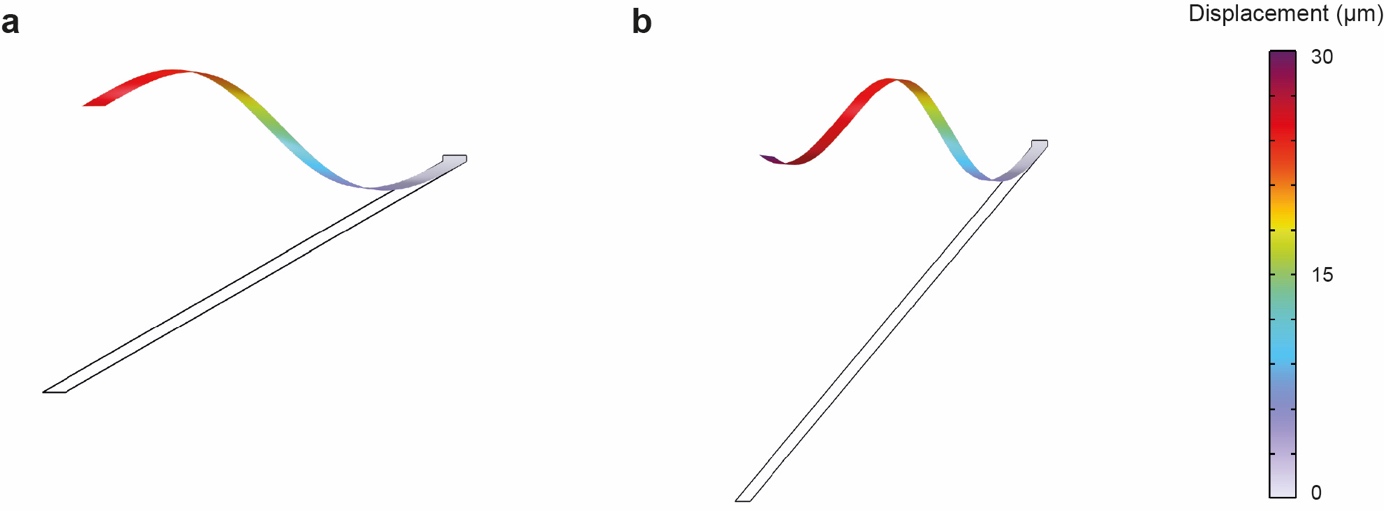


**Figure S7.** Simulation results of helices. (a) 30° and (b) 40°. Note that angles are assigned to 30° and 40°, as a 30° angle corresponds to 60° and a 40° angle to 50°.


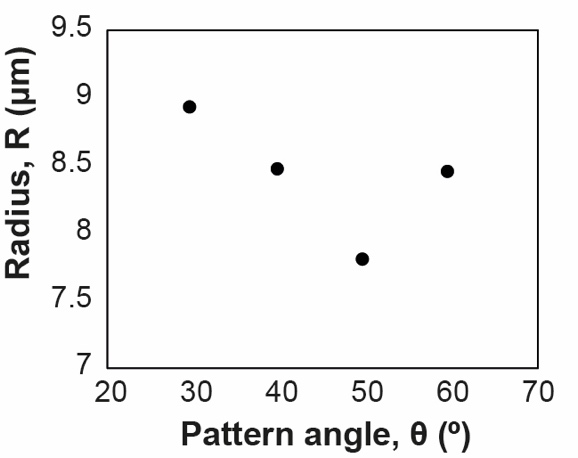


**Figure S8.** Change in helical radius as a function of pattern angles (θ).


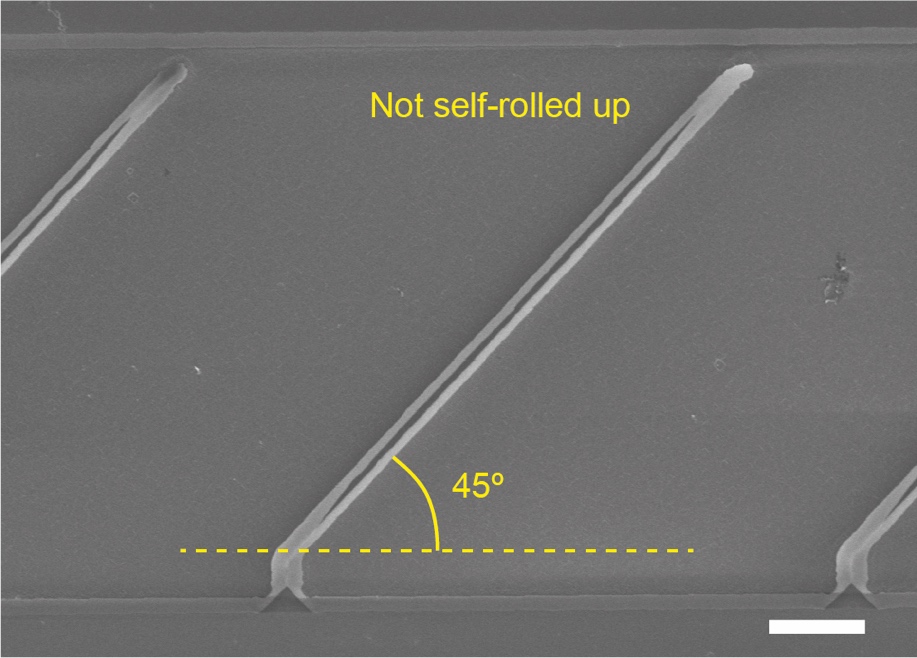


**Figure S9.** The diagonal pattern with an angle (θ) of 45° from the [100] axis. Because of the slow etching rate of MgO along the [110] axis, the 45° pattern was not fully etched while other patterns with angles of 30°, 40°, 50°, and 60° were fully detached from the substrate and formed helices. With enough etching time, the diagonal pattern with the 45° angle formed helices with random chirality (Scale bar: 10 µm).


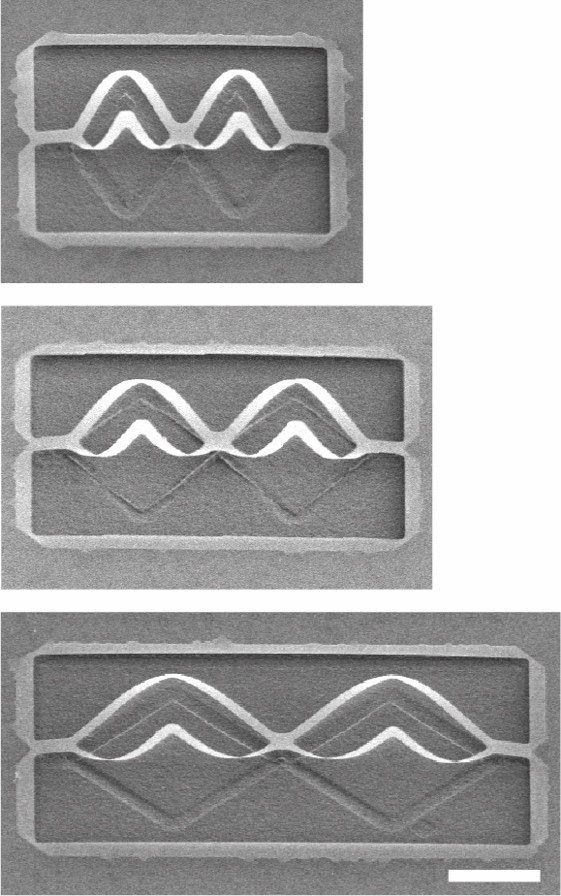


**Figure S10.** SEM images of diamond-kirigami 3-D frames with diamond pattern angles of 60°, 80°, and 100° (45° tilted side view, Scale bar: 10 µm).


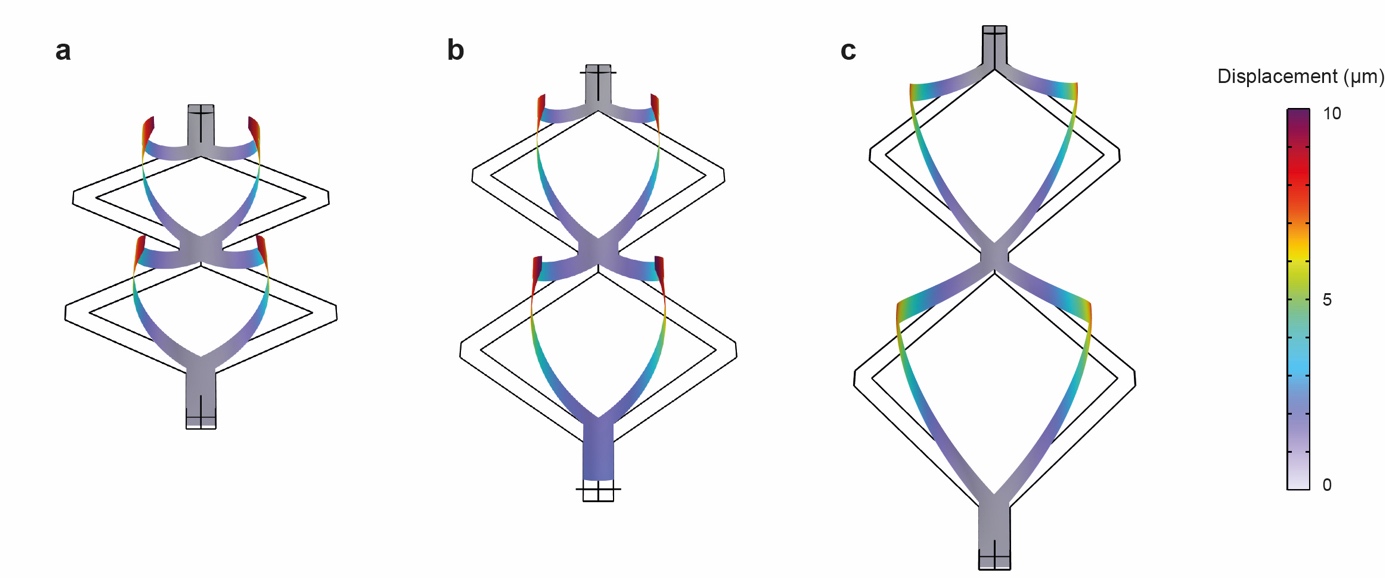


**Figure S11.** Simulation results of diamond-kirigami 3-D frames.


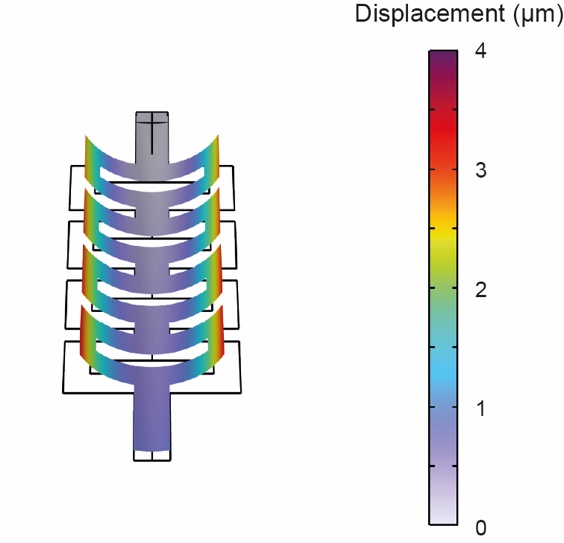


**Figure S12.** Simulation results of ribbon-kirigami 3-D frame.


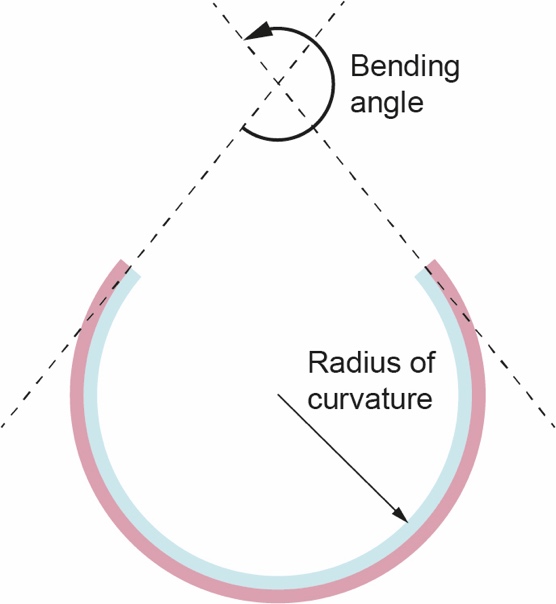


**Figure S13.** Schematic diagram showing how to measure the bending angle of the curved shape (red: cobalt ferrite, blue: barium titanate).


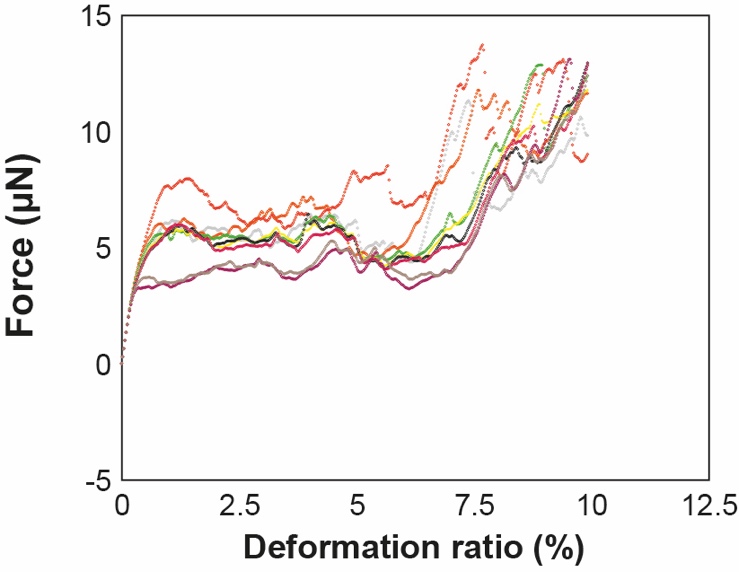


**Figure S14.** Force-displacement curves demonstrating the repeatable behavior of the ribbon-kirigami 3-D frame through tensile testing. In contrast, the diamond-kirigami 3D frames exhibit the same curve with each repetition.


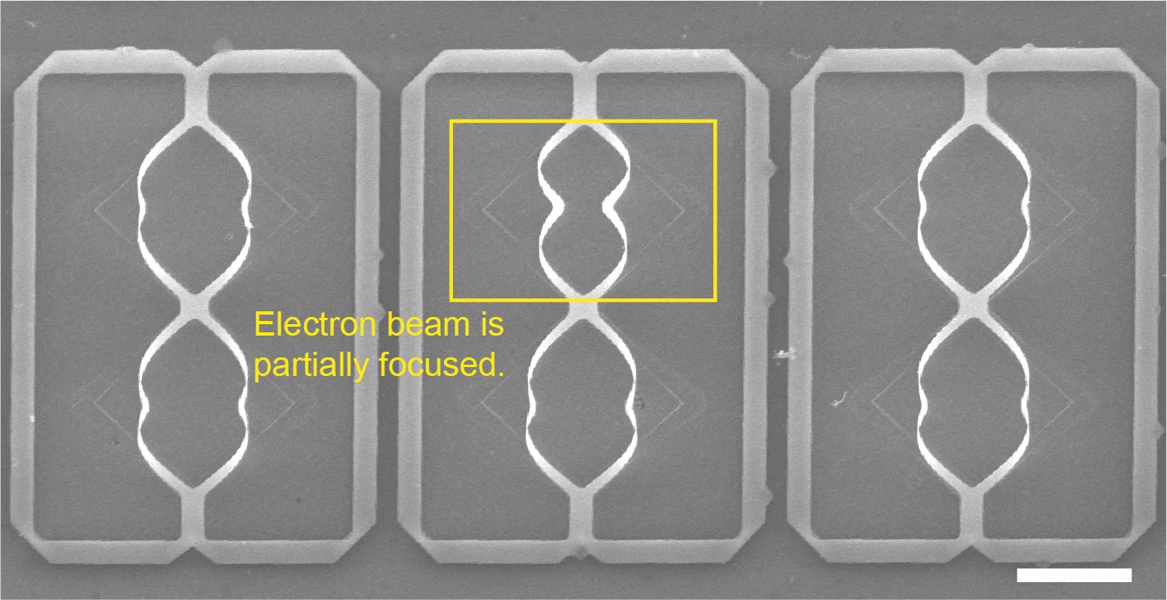


**Figure S15.** Partial deformation of the diamond-kirigami frame by magnifying specific area in SEM (Scale bar: 10 µm).


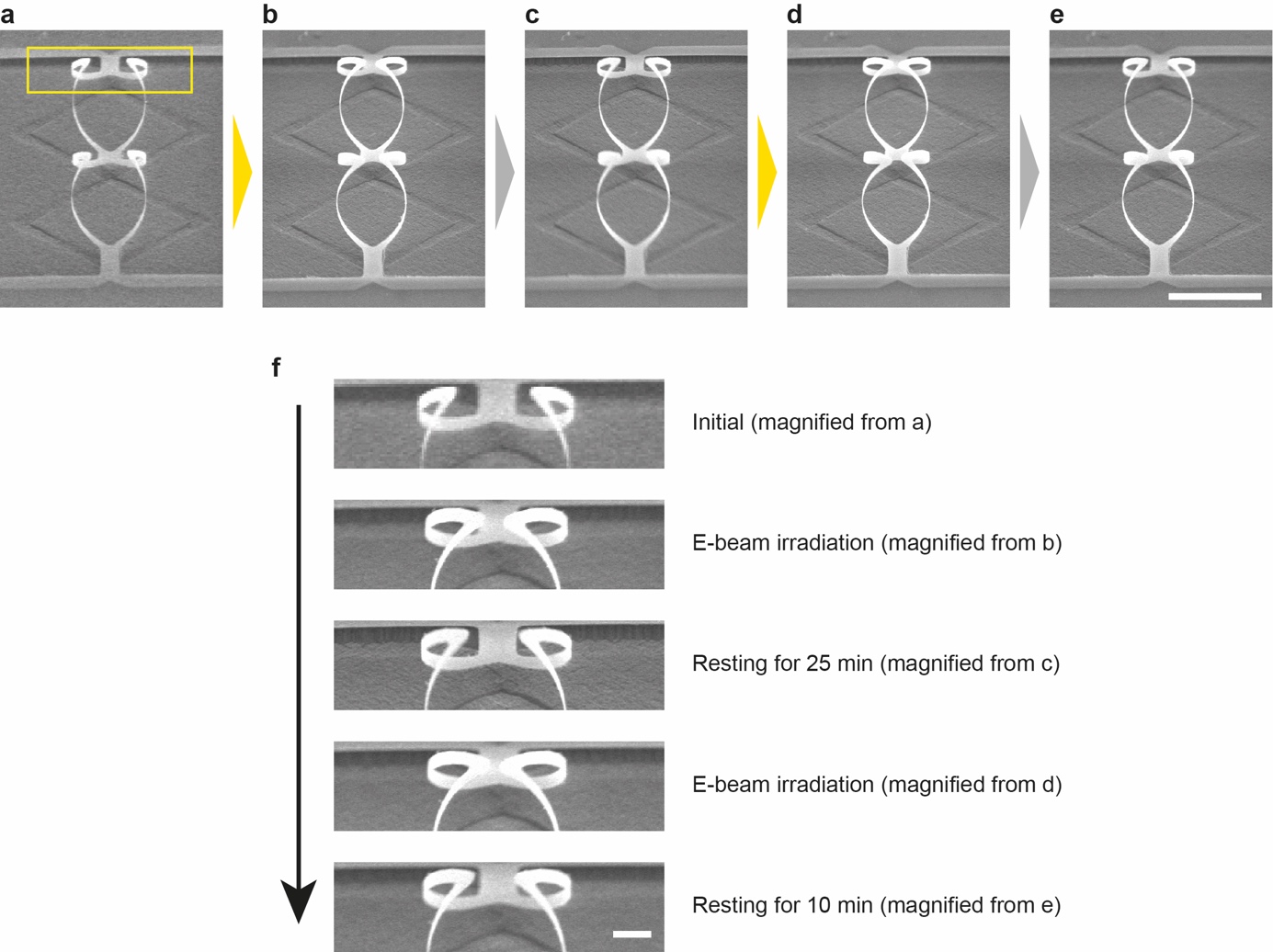


**Figure S16.** Shape-morphing of the diamond-kirigami frame over time with electron beam irradiation and subsequent resting. (a) Initial state. (b, d) Bent by electron beam irradiation. (c, e) Relaxed after resting. (f) Comparison of the shape change, with magnified view (yellow box in (a)) of stages (a-e).


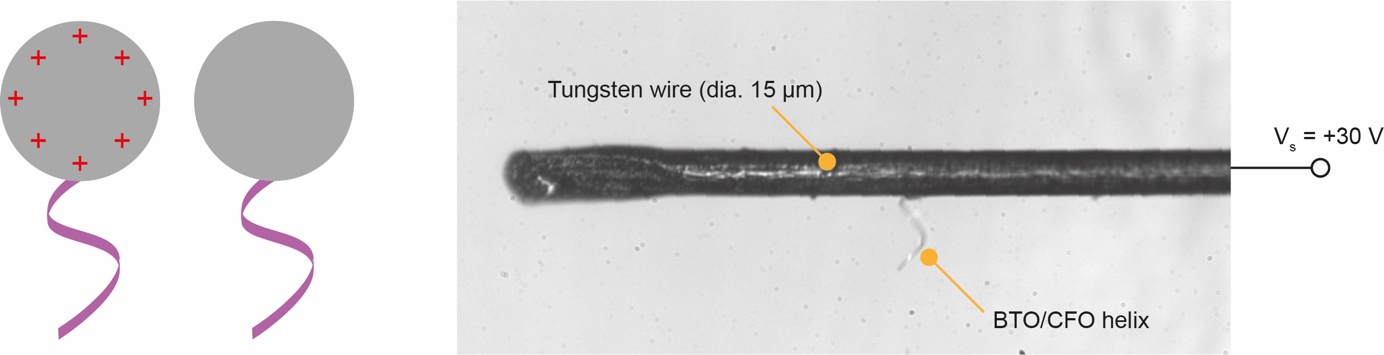


**Figure S17.** (Left) Schematic diagram of cross-sectional sample configuration. (Right) Experimental setup for shape-morphing of BTO/CFO helix via application of low-level electric field outside vacuum chamber.


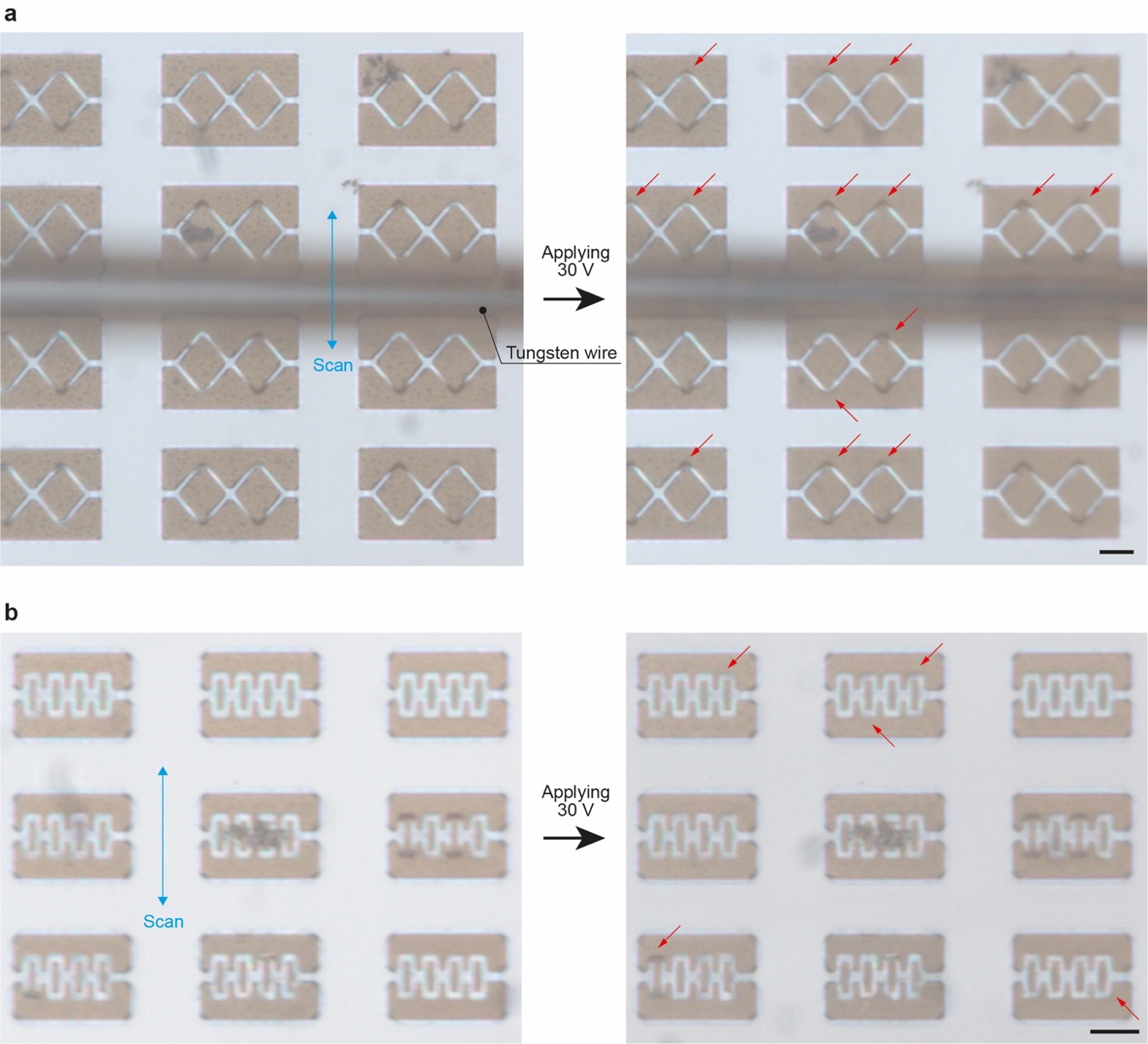


**Figure S18.** Shape-morphing of kirigami 3D-frames induced by the application of an electric potential (+30 V applied to the wire). (a) An 80° diamond-kirigami frame. (b) A ribbon-kirigami frame. Upon applying the electric potential, the wire scanned the structures for 3 minutes. The red arrows highlight the shape changes. Scale bars indicate 10 µm.
